# De-Escalation of Treatment in Women Aged ≥80 Years with Breast Cancer: A Retrospective Analysis from Two Breast Centers

**DOI:** 10.3390/curroncol32090482

**Published:** 2025-08-28

**Authors:** Gianmarco Piccolino, Giulia Cardelli, Francesca Arienzo, Emanuele Zarba Meli, Elena Del Giudice, Leopoldo Costarelli, Rosalinda Rossi, Claudia Scaringi, Tiziana Mastropietro, Laura Broglia, Valeria Vitale, Federica Bergamo, Elena Manna, Massimo La Pinta, Lorenzo Palleschi, Andrea Loreti, Augusto Lombardi, Lucio Fortunato

**Affiliations:** 1 Breast Center, Department of Surgery, San Giovanni-Addolorata Hospital, 00184 Rome, Italy; ezarbameli@hsangiovanni.roma.it (E.Z.M.); tmastropietro@hsangiovanni.roma.it (T.M.); emanna@hsangiovanni.roma.it (E.M.); mlapinta@hsangiovanni.roma.it (M.L.P.); aloreti@hsangiovanni.roma.it (A.L.); lfortunato@hsangiovanni.roma.it (L.F.); 2 Department of Surgical Science, University of Rome “La Sapienza”, Sant’ Andrea Hospital, 00189 Rome, Italy; valeria.vitale@uniroma1.it (V.V.); federica.bergamo@uniroma1.it (F.B.); augusto.lombardi@uniroma1.it (A.L.); 3 Department of Surgical Science, University of Rome, “Tor Vergata” Hospital, 00133 Rome, Italy; giulia.cardelli@ptvonline.it; 4 Pathology Unit, San Giovanni-Addolorata Hospital, 00184 Rome, Italy; francesca.arienzo@uniroma1.it (F.A.); lcostarelli@hsangiovanni.roma.it (L.C.); 5 Geriatric Unit, San Giovanni-Addolorata Hospital, 00184 Rome, Italy; edelgiudice@hsangiovanni.roma.it (E.D.G.); lpalleschi@hsangiovanni.roma.it (L.P.); 6 Department of Oncology, San Giovanni-Addolorata Hospital, 00184 Rome, Italy; rrossi@hsangiovanni.roma.it; 7 Radiotherapy Unit, San Giovanni-Addolorata Hospital, 00184 Rome, Italy; cscaringi@hsangiovanni.roma.it; 8 Breast Radiology, San Giovanni-Addolorata Hospital, 00184 Rome, Italy; lbroglia@hsangiovanni.roma.it

**Keywords:** older adults, breast cancer, surgery

## Abstract

Background: Breast cancer is frequently diagnosed in older women. However, the impact of surgery on survival is not well studied and prognosis for women ≥ 80 years of age is progressively depending on comorbidities. Methods: Medical records of consecutive women aged ≥ 80 years diagnosed with primary breast cancer treated with upfront surgery at two Breast Centers from 2011 to 2021 were retrospectively analyzed. Results: A total of 553 consecutive women with a median age of 83 years and a median tumor diameter of 21 mm were analyzed (574 lesions). Clinical Stages II or III were found in 263/574 (46%) and 101/574 cases (18%), respectively. Axillary staging was completely omitted for 94/542 invasive lesions (17%), and this increased over time from 2% to 33% (*p* < 0.001). Adjuvant hormone therapy and radiotherapy were omitted in 134/490 (27%) and in 122/420 patients (29%), respectively, while only 26/195 (13%) of patients with a clear clinical indication received adjuvant chemotherapy. At a median follow-up of 61 months (6–147) the 5- and 10-years overall survival (OS) were 64% and 21%, while breast cancer-specific survival (BCSS) at 5 and 10 years were 94% and 78%, respectively. Adjuvant therapies were not associated with a significant improvement in BCSS, while worse OS was associated with older age or more comorbidities as measured by the Charlson Comorbidity Index (CCI) (*p* < 0.001 and *p* = 0.012, respectively). Conclusions: Breast surgery, when possible, has a primary role even for women > 80 years of age, and it is associated with a reasonable BCSS. De-escalation of adjuvant therapies should be considered in this setting because survival is largely determined by age and co-morbidities.

## Simple Summary

The impact of surgery and adjuvant therapies on survival for women ≥80 years of age is not well quantified, because these patients more often have multiple co-morbidities and are at risk of complications. We describe one of the largest institutional experiences in this setting and report a 94% 5-year breast cancer-specific survival (BCSS), suggesting a prominent role of surgery, if technically feasible, while adjuvant hormone therapy, radiotherapy, and chemotherapy were frequently omitted and were not significantly associated with an improvement in BCSS.

## 1. Introduction

Worldwide, breast cancer is the most common malignant neoplasm in women, and age represents one of the main risk factors. More than 30% of breast cancer patients are currently over 70 years of age at the time of diagnosis [1]. While the global population is rapidly aging, the proportion of adults > 65 years old is increasing from 6% (in 1990) to 9% (in 2019), and this percentage is estimated to further expand to 16% by the year 2050, especially in the western countries and South-East Asia [2].

It is well known that an advanced stage of disease is more common among older women, in part because they are excluded from current screening strategies [3]. At the same time, most randomized clinical trials on breast cancer exclude women > 80 years of age, and therefore, little is known about the effectiveness of treatments considered standard for other age groups [4]. Finally, these patients are more often at risk of complications following surgical and oncological treatments, with a potentially negative impact on quality of life and the possibility to carry out daily living functions [5].

The definition of “older adults” is becoming progressively elaborated and their management may represent a challenge for clinicians. In fact, many of these patients enjoy a good quality of life, but at the same time individual fragility becomes increasingly common with aging [5]. Therefore, the decision process is complex with risk of both “under-treatments” and possible increase in the risk of loco-regional and systemic recurrence [6] and “over-treatments”, because therapies may not be strictly necessary to impact on survival [7]. In this context, a multidisciplinary geriatric assessment (GA) has been recommended as a key factor to determine patient’s physiological age and guide a shared multidisciplinary therapeutic intervention [8].

The impact of surgery on survival for women > 80 years old has not been well studied with a paucity of institutional or large national reports. Although prospective or randomized clinical for this age group trials are lacking, treatment de-escalation has been suggested, in general, for these patients and it is included in the guidelines. Two systematic reviews have shown that both local control and survival are better with primary surgery compared to primary endocrine therapy with Tamoxifen, although in one large cohort study no clear differences were reported [9,10,11]. Both the Society of Surgical Oncology (SSO) and the American Board of Internal Medicine (ABIM) have questioned the role of axillary staging since 2016 with the “Choosing Wisely Campaign” [12], on the bases of several prospective trials reporting no advantage in this group of patients on loco-regional recurrence and breast cancer-specific survival (BCSS) [7,13,14,15]. However, these studies had limitations, as CALGB 9343 did not randomize patients for surgical axillary treatment but for breast irradiation. Apart from this contest, recent randomized trials (SOUND and INSEMA) have shown that omission of axillary staging is non-inferior to sentinel lymph node biopsy for patients with tumors < 2 cm and a negative axillary ultrasound [16,17]. In addition, both the NCCN and NICE guidelines allow the omission of adjuvant radiotherapy after breast-conserving surgery (BCS) in this contest for women with clinically negative nodes and luminal-like tumors (HR+/HER2−) [18,19].

The aim of this study is to describe a “real world” practice and to evaluate overall survival (OS) and BCSS at 5 and 10 years among women > 80 years who underwent surgery for primary breast cancer at two accredited Breast Centers in Rome (Italy), and to further define possible de-escalation strategies for these women.

## 2. Materials and Methods

The study received the approval of the Lazio-2 Ethics Committee (Protocol N. 0078775/2023, 21 April 2023).

Medical records of consecutive women ≥ 80 years diagnosed with primary breast cancer operated at two major Breast Centers in Rome (San Giovanni-Addolorata Hospital and Sant’ Andrea University Hospital) from January 2011 to December 2021, were analyzed retrospectively from a prospectively maintained database. Both Breast Centers are accredited by the Lazio Regional Network. The San Giovanni-Addolorata Hospital received the BCCERT/ITALCERT Breast Certification (based on EUSOMA indicators and recommendations) [20] since 2017, confirmed in 2024. Patient’s data were prospectively maintained onto DATABREASTTM, a certified database with more than 600 items for each patient. Male patients, women with second breast lesions or recurrences, and patients who underwent any form of neoadjuvant therapy were excluded from the study. Two patients with disease stage IV who were operated on for locally advanced disease and control of symptoms (ulceration or bleeding) were excluded.

Patients were treated after multidisciplinary evaluation, considering their age and comorbidities, tumor characteristics, stage of disease, and patient’s personal preferences.

Anatomical and Pathologic Stages were defined according to the last TNM staging system (AJCC, Eighth Edition) [21]. The “Prognostic” stage was calculated for all patients treated with complete surgical excision and nodal staging, requiring analysis of grade, estrogen and progesteron receptor status (ER, PR) (positive/negative) and HER2 receptor status (positive/negative) [22].

Follow-up was performed with periodic clinical breast examination, mammography, and breast ultrasound if requested by the examining radiologist and according to breast density (BIRADS). Systemic radiologic investigations were usually kept at minimum, unless symptoms developed. Patients who completed the first 5 years of follow-up or patients who did not come to visit for personal reasons were contacted by phone or email, as per standard of care in our Breast Centers.

Invasive breast cancers (IBCs) were divided into 3 groups based on hormone receptor (HR) and Human Epidermal Growth Factor Receptor 2 (HER2) positivity into HR+/HER2− (Luminal-like), HER2+, and triple negative (TN) [23].

The patients were divided into three groups based on age: 80–84, 85–89, and ≥90 years of age. Comorbidities were identified and classified according to the Charlson Comorbidity Index (CCI) [24]. Subsequently, depending on the CCI score, they were classified into three groups: mild comorbidity (CCI 0–2), moderate comorbidity (CCI 3–4), and severe comorbidity (CCI ≥ 5).

Patients were not routinely referred for a full geriatric assessment, although this is now required after identification of low scores at standard initial evaluation including a Mini-cog (cognitive) and SPPB (physical) test administered at first clinical presentation [25,26].

The primary outcomes were OS and BCSS at 5- and 10-years. The main predictors considered were age, CCI group, tumor size, nodal stage, prognostic stage, type of surgery (M vs. BCS), histological and molecular subtypes, and adjuvant therapies. Continuous values were summarized using median and range, whereas categorical variables were presented as absolute frequencies and percentages. Chi-square analyses were employed to compare categorical variables. We conducted the Kaplan–Meier survival analysis to assess the survival probability of the population for OS and BCSS at any given time, and the log-rank test to compare survival probabilities among groups. The null hypothesis of the test is that there is no difference between the survival probabilities at any time point, and the alternative hypothesis states that a survival difference exists. A *p*-value of less than 0.05 is statistically significant, signifying robust evidence against the null hypothesis. To assess the association between individual covariates and OS and BCSS, univariate analyses were performed using Cox proportional hazards regression models.

Variables that were statistically significant in the univariate analysis (age, CCI group, prognostic stage, pN, pT, radiotherapy, and surgery) were subsequently included in multivariate Cox proportional hazards regression models to simultaneously assess their independent association with the risk of death over time. We report the results of univariate and multivariate analyses as hazard ratio (HR), 95% confidence interval (IC), and *p*-value. An HR of 1 indicates no effect; an HR greater than 1 indicates a covariate that is positively associated with the event probability; an HR less than 1 suggests a decrease in hazard. Statistical analysis was conducted utilizing GraphPad Prism, version 10.4.1, GraphPad Software, San Diego, CA, USA^®^.

## 3. Results

In the study period, 553 patients were analyzed. Bilateral breast cancer was found in 21 patients (3.8%), for a total of 574 lesions included. The median age was 83 years (80–99), and the median tumor diameter was 21 mm (1–110). A palpable lesion was reported in 456/574 tumors (79.5%). Tumor characteristics are summarized in Table 1. Multifocality or multicentricity was diagnosed in 19/553 cases (7%). A family history of breast cancer was recorded in 153/553 women (28%).

Breast-conserving surgery (BCS) or mastectomy were performed in 390/574 (68%) and 184/574 (32%) lesions, respectively. Axillary staging was completely omitted in 94/542 IBCs (17%), and this increased during the periods 2011–2016 and 2017–2021, from 5/201 cases (2%) to 112/341 (33%), respectively (*p* < 0.001). Overall, axillary staging was omitted for BCS and mastectomy in 78/359 (22%) and 16/183 (9%), respectively (*p* < 0.001).

SLNB only and axillary lymph node dissection (ALND) were performed in 274/542 (51%) and in 110/542 IBCs (20%), respectively. A completion ALND was performed after a positive SLNB in 64/574 cases (12%).

Adjuvant hormone therapy was omitted in 134/490 patients with HR+ tumors (27%). Only 26/195 (13%) of patients with prognostic stage II–III tumors received adjuvant chemotherapy (26/521 patients with invasive disease = 4.9%). Adjuvant chemotherapy for triple negative (TN) and HER2+ tumors was prescribed more frequently, in 4/35 (11%) and in 9/53 patients (17%), respectively (*p*< 0.001).

Radiotherapy was omitted in 122/420 patients after BCS (29%).

**Figure 1:**
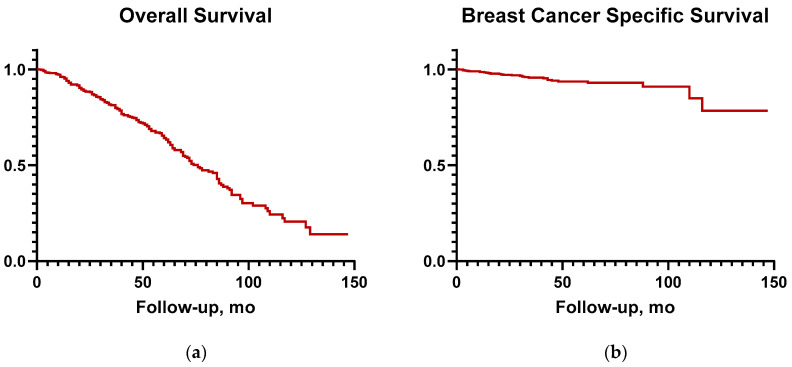
(**a**) Overall survival; (**b**) breast cancer-specific survival.

The median follow-up was 61 months (6–147). The median OS was 76 months (12–147). The OS at 5 and 10 years were 64% and 21%, respectively (Figure 1a). The 5- and 10-year BCSS were 94% and 78%, respectively (Figure 1b).

**Figure 2:**
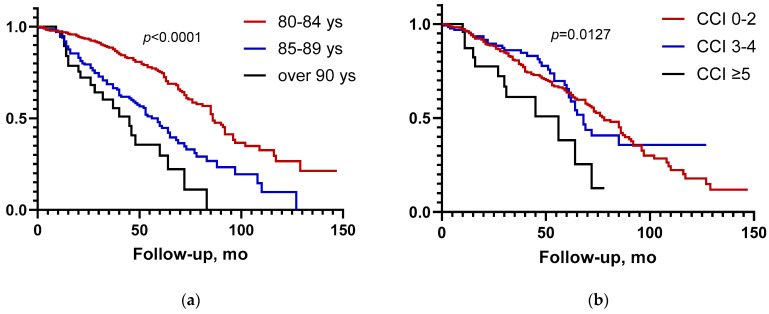
(**a**) Overall survival according to age group; (**b**) overall survival according to comorbidities groups.

After stratification by age groups (80–84 years, 354/553, 64%; 85–89 years, 158/553, 29%; >90 years, 41/553, 7%), a statistically significant difference in terms of median OS was found (86 vs. 60 vs. 40 months, respectively; *p* < 0.001) (Figure 2a).

After stratification by CCI score, the degree of comorbidity (mild, group 1: CCI 0–2, 411/574 lesions 71.5%; moderate, group 2: CCI 3–4, 138/574 24%; severe, group 3: CCI ≥ 5, 25/574, 4.5%), was associated with a significant difference in median OS (group 3 vs. groups 1 and 2, 76 vs. 56 months; *p* = 0.0127) (Figure 2b).

Pathological tumor stage (pT 0–1 vs. ≥ pT2) and nodal stage (pN 0–1 vs. ≥pN2) were significantly associated with OS (*p* < 0.0001 and *p* = 0.0026, respectively) and BCSS (*p* = 0.0038 and *p* < 0.0001, respectively). No statistically significant differences in OS were found among histological subtypes or biological subgroups, as defined by immunohistochemistry (*p* = 0.19).

**Figure 3:**
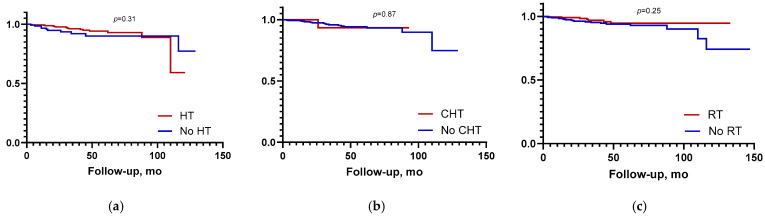
Breast cancer-specific survival according to implementation of adjuvant therapies. (**a**) hormone therapy (HT); (**b**) chemotherapy (CHT); (**c**) radiotherapy (RT).

The omission of adjuvant treatment with hormone therapy, chemotherapy, or radiotherapy did not significantly impact BCSS (*p* = 0.31, *p* = 0.87, *p* = 0.25) (Figure 3).

Age, CCI group, pT, pN, and adjuvant RT significantly influenced OS, according to univariate analysis. All the above variables were confirmed as prognostic factors in the multivariate analysis, except for nodal stage (*p* = 0.1430) (Table 2).

Age and comorbidities were not independent prognostic factors for BCSS. In univariate analysis, BCSS was significantly associated with tumor size, nodal stage, pathological stage, and type of surgery (mastectomy vs. BCS). However, only nodal stage ≥ 2 was confirmed as a significant prognostic factor in multivariate analysis for BCSS (Table 3).

## 4. Discussion

This is one of the largest institutional studies with focus on breast cancer surgery for women ≥ 80 years old. We found that, at a median follow-up of 61 months (6–147), the 5-year OS was 64% while 21% of patients surgically treated survived at 10 years. This specific group of patients enjoyed a considerable BCSS of 5 and 10 years (94% and 78%, respectively) suggesting that surgery should be considered as a first option, if co-morbidities do not contraindicate this choice. In a small retrospective comparison between surgery and primary endocrine therapy for patients aged ≥ 80 years, the former was found to offer significantly better results in terms of 5-years survival rates and significant decrease in local–regional recurrence [27]. More recently, a retrospective cohort study among 113 women > 90 years showed a higher OS and a lower breast cancer related mortality in patients treated with surgery (77/113) compared with non-surgical patients [28]. For women > 70 years of age, a Cochrane review of seven clinical studies including 1571 women, and a systematic review of 6 controlled trials and 31 non-randomized studies have reported that primary surgery has a better progression-free survival compared with primary medical treatment, particularly in the case of a life expectancy of five years or more [9,10]. In the same age group, a recent prospective, multicenter, observational study on 2854 ER+ breast cancer women from UK, recruited from 56 breast centers, reported that for the majority of these women, surgery is oncologically superior to primary endocrine therapy, and that treatment should be individualized for those who are very old or frail with a life expectancy of less the five years [29].

Omission of axillary staging, for cN0 women, has been suggested as an integral part of the surgical strategy for “older women”, since the Society of Surgical Oncology (SSO) adopted the “Choosing Wisely Campaign” in 2016 for patients with luminal-like tumors based on several studies showing no impact in this group of patients on loco-regional recurrence and BCSS [7,13,14,15]. In fact, a Canadian study among 2662 women aged ≥ 70 years with luminal-like (HR+/HER2−) tumors showed that breast cancer-specific survival (BCSS) was very high (96%) if patients received adjuvant hormone therapy, regardless of the sentinel lymph node biopsy (SLNB) result [30]. To overcome the lack of controlled trials on omission of axillary staging, the SOUND trial (SLNB vs. Observation after axillary Ultra-Sound) randomized 1405 women with T1 tumors and cN0 on preoperative axillary ultrasound (average age of approximately 60 years, over 75% in peri-post menopause) and recently demonstrated that surgical axillary observation was not inferior to SLNB in terms of distant DFS (98% vs. 97.7%) and local control [16]. Results of another recent non-inferiority, randomized trial from Germany and Austria on 4858 patients (INSEMA) corroborated this finding [17]. It is important to emphasize that these data do not only apply to “older adults”, but rather to all “eligible” patients.

Although the rate of omission of axillary staging in our cohort was still quite low (17%), this is somewhat in line with previous reports indicating that until recently, most “older women” have undergone SLNB, and in a US dataset, omission of lymph node staging has been reported in only 23% of cases [31]. However, we found a progressive increase in SLN omission from 2% to 33%, indicating that in our experience, surgeons are progressively aligned with the current understanding that de-escalation may limit morbidity without compromising outcome. More recently, a recent report from the University of Pittsburgh Health System in 2021, on 2195 HR+/HER2− women aged ≥70 years has confirmed that SLNB had no significant impact on loco-regional control and DFS after propensity score-match [32]. In addition, in this study, a low incidence of positive sentinel lymph nodes was found (11.5%), and patients with a positive SLN did not show a worse DFS or loco-regional control at multivariate analysis.

In the last 15 years, excellent outcomes after primary surgery in the setting of age > 80 years are confirmed by six other institutional reports on a total of 985 patients [27,33,34,35,36,37] and three large database studies indicating that this is a debated issue, with a special clinical interest (Table 4).

In 2022, a large study from the National Cancer Database (NCDB, USA) on a total of 59,043 women with stage I–III tumors, confirmed good results after primary breast surgery with a median OS of 6.7 years [31]. Similarly, a report from the Alberta Cancer Registry (Canada) on a total of 1369 women in 2021 found that surgery in this setting is associated with a 3-years OS and BCSS of 66% and 70.5%, respectively [39]. Finally, a large study from the Netherlands Cancer Registry (NRC) in 2020 on 6464 women with stage I–II, HR+ tumors, demonstrated that volume of breast surgery (high vs. low number of cases) is associated with higher OS and BCSS, suggesting that a special expertise and dedication is required to meet all special needs for these vulnerable women [38].

In the setting of ductal cancer in situ, a recent meta-analysis of five studies among women over the age of 75 years has examined real-world practice patterns, concluding that therapeutic strategies should be tailored on life expectancy, comorbidities, and performance status [40].

We report that different age groups and higher comorbidity scores resulted in statistically different OS, underlining the need to individualize treatments. Therefore, screening for frailty and geriatric assessment are becoming an integral part of multidisciplinary assessment to better delineate competing causes of mortality, as evidenced by a policy review and a conjoint EUSOMA-SIOG consensus statement (European Society of Breast Cancer Specialists and the International Society of Geriatric Oncology) [41]. In particular, surgery for women with age > 90 years or with a CCI > 5 should receive a carefully individualized assessment and management, as their median survival in our cohort was limited and below 5 years.

Interestingly, in our study only nodal stage > 2 was associated with BCSS at multivariate analysis, and adjuvant hormone therapy; chemotherapy or radiotherapy were not associated with significant improvements, suggesting that these treatments should be discussed case by case, considering the eventual co-morbidities and life expectancy. Perhaps, in this age group, while advanced stage retains an impact on outcome, competing factors, such as age and comorbidities, dilute potential benefits of adjuvant therapies. This is probably the reason why neoadjuvant chemotherapy was seldom selected in this “real world” experience, even for patients with a prognostic stage II–III.

In line with this strategy, the National Comprehensive Cancer Network (NCCN) guideline has indicated that omission of radiotherapy may be considered for older women with early-stage disease, if hormone therapy is indicated. This is consistent with a meta-analysis of five studies (including two trials, CALGB-9343 and PRIME II), involving a total of 3776 post-menopausal women, which showed a benefit in terms of local recurrence without a significant impact on overall survival [15,42,43]. Despite all that, de-escalation is far from a consistent implementation, and rates of radiotherapy continue to be high [44,45].

Data regarding omission of hormone therapy (HT) and chemotherapy in this setting are even more limited. Our study, in line with other reports [46], confirms a high percentage (83%) of HR+/HER2− tumors in women ≥ 80 years old. However, tumor characteristics in older adults sometimes have a “paradoxical” feature and a retrospective analysis performed on 3947 women, reclassified according to intrinsic molecular subgroups with gene expression microarray dataset (PAM50), showed that luminal B-like tumors were twice as common among patients > 70 years of age if compared to women in the pre-menopausal group (<50 years of age) (32% vs. 15%), while in the same age groups the combined incidence of HER2+ or TN tumors were 14% and 38%, respectively [47]. A retrospective cohort study on 420 women aged ≥ 70 years, stage I–III, showed that the impact of chemotherapy on BCSS is not significant among women ≥ 75 years of age [48].

Therefore, while optimal treatment strategy in this contest remains uncertain, the EUROPA Trial is a phase 3 non-inferiority, randomized multicentric study for older women ≥ 70 years, with stage I, luminal-A like tumors, aiming to compare HT-alone with partial breast irradiation (PBI)-alone, after BCS [49]. Preliminary results were recently published and showed that endocrine therapy is associated with a greater reduction in quality of life.

This study has several limitations. First, being a retrospective study performed in two Breast Centers, it is subject to selection bias. We have no data on patients who were treated or observed without surgery in this setting. The second limitation is the quality of the follow-up beyond the first 5 years after surgery, because clinical examinations and mammography were no longer carried out in most cases, and data has been collected by telephone interview or email enquiry, often with the relatives of the deceased patients. Third, particularly among patients operated in the first study period, insufficient data was collected regarding the type of radiotherapy and the effective adherence to adjuvant systemic therapies. Finally, we have no data on geriatric evaluation, morbidity of treatments, or quality of life assessment, and these issues would be particularly relevant nowadays for a better understanding of this issue.

However, as no randomized trials have been performed for this age group, the strength of this experience is represented by the large number of women studied, compared to previous institutional reports, with a long-term follow-up by two dedicated Breast Centers with a prospectively maintained database.

## 5. Conclusions

Breast surgery for selected patients > 80 years of age is associated with a reasonable OS and BCSS at 5 years, and therefore surgery remains a first choice of treatment in this setting. De-escalation of nodal staging for cN0 is feasible and is not associated, in our retrospective analysis, with detrimental results.

Adjuvant hormone therapy and radiotherapy in our study were omitted in more than 1 in 4 women, while 9 out of 10 women, with a clear clinical indication for advanced disease, did not undergo chemotherapy.

De-escalation of adjuvant therapies among these women might be considered in selected cases, since they may not be associated with significant improvements in terms of survival. This remains a challenging decision, and these options should be discussed with the patients taking into account their wishes and values, which may be quite variable.

## Figures and Tables

**Table 1:** Tumor characteristics.

Total (N = 574)	N (%)
Histopatological Type	
IDC (NST)	439 (76)
ILC	74 (14)
DCIS	32 (5)
Other	29 (5)
Tumor T Stage	
pTis	32 (5)
pT1	266 (47)
pT2	133 (23)
pT3–4	143 (25)
Nodal Stage	
pNx	111 (19)
pN0	228 (40)
pN1	142 (25)
pN2–3	93 (16)
**Invasive Breast cancer (IBC) (N = 542)**	
Molecular Receptors Type	
HR+/HER2−	454 (83)
HER2+	53 (10)
TNBC	35 (7)
Lymphovascular invasion	
Yes	120 (22)
Grading	
G1	82 (15)
G2–G3	460 (85)
Clinical Stage	
I	178 (33)
II	263 (48)
III	101 (19)
Prognostical Stage	
N/A *	94 (17)
I	253 (47)
II	101 (19)
III	94 (17)

IBC, invasive breast cancer, IDC, invasive ductal carcinoma; DCIS, ductal carcinoma in situ; ILC, invasive lobular carcinoma; HR+/HER2−, luminal-like carcinoma; HER2+, Human Epidermal Growth Factor Receptor 2-positive carcinoma; TNBC, triple negative breast carcinoma. * N/A, not calculable because lymph node staging was not performed.

**Table 2:** Univariate and multivariate analysis for overall survival (OS).

	Univariate Analysis			Multivariate Analysis		
Variable	HR	95%CI	*p*	HR	95%CI	*p*
Age ≥ 85 ys	2.227	1.680–2.928	<0.0001	1.701	1.204–2.397	0.0025
CCI group I–II vs. III	2.340	1.299–4.033	0.0047	2.276	1.007–4.458	0.0280
Tumor Size ≥ pT2	2.084	1.576–2.768	<0.0001	1.798	1.268–2.578	0.0012
Nodal Stage ≥ pN2	1.755	1.203–2.506	0.0026	1.328	0.8989–1.926	0.1430
RT vs. No RT	2.354	1.683–3.373	<0.0001	0.6111	0.4071–0.8966	0.0142

ys, years; CCI, Charlson Comorbidity Index; RT, radiotherapy.

**Table 3:** Univariate and multivariate analysis for breast cancer-specific survival (BCSS).

	Univariate Analysis			Multivariate Analysis		
Variable	HR	95%CI	*p*	HR	95%CI	*p*
Tumor Size ≥ pT2	3.367	1.535–8.140	0.0038	1.683	0.5877–5.699	0.3625
Nodal Stage ≥ pN2	5.497	2.370–13.03	<0.0001	2.906	1.166–7.620	0.0240
Prognostic Stage ≥ II	6.444	2.404–22.31	0.0008	2.700	0.7822–11.36	0.1404
Surgery (M vs. BCS)	2.852	1.355–6.169	0.0061	1.492	0.6124–3.787	0.3831

M, mastectomy; BCS, breast-conserving surgery.

**Table 4:** Tumor summary of studies published in the last 15 years regarding the effectiveness of breast surgery in women aged ≥80 years.

Ref.	Year	Country	Study	N	Results
[33]	2011	USA	Cyr A et al., 2011. J Surg Oncol.	134	BCSS (5 y): 83%
[34]	2013	Spain	Cortadellas T et al., 2013. Int J Surg.	175	Early stage median BCSS: 9 y. Locally advanced median BCSS: 6.3 y.
[35]	2014	Slovenia	Besic N et al., 2014. BMC Cancer.	154	BCSS (5 y): 83%
[36]	2019	Finland	Ojala K et al., 2019. Eur J Surg Oncol.	401	BCSS (5 y): 82% OS (5 y): 51%
[38]	2020	Netherlands	De Boer AZ et al., 2020. Br J Surg. NCR.	6464 Stage I–II, HR+/HER2−	BCSS (5 y): 90.2–92% (10 y): 71–88% OS (5 y): 48.3–51% (10 y): 15–20%
[37]	2020	Switzerland	Di Lascio S et al., 2021. EJSO.	44 Age ≥89	Median OS: 4,1 y
[39]	2021	Canada	Al-Rashdan A et al., 2021. The Breast; Alberta Cancer Registry.	1369	BCSS (3 y): 70.5% OS (3 y): 66%
[31]	2022	USA	Frebault J et al., 2022. Clin Breast Cancer; NCDB.	59,043 Stage I–III	Median OS: 6.7 y
[28]	2024	Italy	Ferrucci M et al., 2024. Ann Surg Oncol.	77 Age ≥ 90	OS (5 y): 51%
Present Study	2025	Italy	Piccolino G et al., 2025.	553	Median OS: 6.3 y BCSS (5 y): 94% (10 y): 78% OS (5 y): 64% (10 y): 21%

## Data Availability

The original contributions presented in this study are included in the article. Further inquiries can be directed to the corresponding author.
